# Chemical Analysis of the Antihyperglycemic, and Pancreatic α-Amylase, Lipase, and Intestinal α-Glucosidase Inhibitory Activities of *Cannabis sativa* L. Seed Extracts

**DOI:** 10.3390/molecules29010093

**Published:** 2023-12-22

**Authors:** Salima Haddou, Amal Elrherabi, El Hassania Loukili, Rhizlan Abdnim, Asmae Hbika, Mohamed Bouhrim, Omkulthom Al Kamaly, Asmaa Saleh, Abdelaaty A. Shahat, Mohamed Bnouham, Belkheir Hammouti, Abdelkrim Chahine

**Affiliations:** 1 Laboratory of Advanced Materials and Process Engineering, Faculty of Science, University Ibn Tofail, University Street, B.P. 242, Kenitra 14000, Morocco; salima_haddou@hotmail.fr (S.H.); abdelkrim.chahine@uit.ac.ma (A.C.); 2 Laboratory of Bioresources, Biotechnology, Ethnopharmacology and Health, Faculty of Sciences, University Mohammed 1st, Bd. Med VI B.P. 717, Oujda 60000, Morocco; amal.rhe96@gmail.com (A.E.); r.abdnim@ump.ac.ma (R.A.); mbnouham@yahoo.fr (M.B.); 3 Laboratory of Applied Chemistry & Environment, Faculty of Sciences, University Mohammed 1st, Bd. Med VI B.P. 717, Oujda 60000, Morocco; hassania-loukili@hotmail.com (E.H.L.);; 4 Euro-Mediterranean University of Fes (UEMF), B.P. 15, Fes 30070, Morocco; hammoutib@gmail.com; 5 Laboratories TBC, Laboratory of Pharmacology, Pharmacokinetics and Clinical Pharmacy, Faculty of Pharmacy, University of Lille, 59000 Lille, France; 6 Laboratory of Biological Engineering, Team of Functional and Pathological Biology, Faculty of Sciences and Technology, University Sultan Moulay Slimane, Beni Mellal 23000, Morocco; 7 Department of Pharmaceutical Sciences, College of Pharmacy, Princess Nourah bint Abdulrahman University, P.O. Box 84428, Riyadh 11671, Saudi Arabia; omalkmali@pnu.edu.sa (O.A.K.); asali@pnu.edu.sa (A.S.); 8 Department of Pharmacognosy, College of Pharmacy King Saud University, Riyadh 11362, Saudi Arabia

**Keywords:** *Cannabis sativa* L., α-amylase, lipase, α-glucosidase, postprandial glycemia, HPLC-DAD

## Abstract

Cannabis is considered (*Cannabis sativa* L.) a sacred herb in many countries and is vastly employed in traditional medicine to remedy numerous diseases, such as diabetes. This research investigates the chemical composition of the aqueous extracts from *Cannabis sativa* L. seeds. Furthermore, the impact of these extracts on pancreatic α-amylase and lipase, and intestinal α-glucosidase enzymes is evaluated, as well as their antihyperglycemic effect. Analysis of the chemical composition of the aqueous extract was conducted using high-performance liquid chromatography with a photodiode array detector (HPLC-DAD). In contrast, the ethanol, hexanic, dichloromethane, and aqueous extract compositions have been established. Additionally, the inhibitory effects of ethanolic, dichloromethane, and aqueous extracts on pancreatic α-amylase and lipase, and intestinal α-glucosidase activities were evaluated in vitro and in vivo. The results of HPLC analysis indicate that the most abundant phenolic compound in the aqueous cannabis seed extract is 3-hydroxycinnamic acid, followed by 4-hydroxybenzoic acid and rutin acid. Moreover, administration of ethanolic and aqueous extracts at a dose of 150 mg/Kg significantly suppressed postprandial hyperglycemia compared to the control group; the ethanolic, dichloromethane, and aqueous extracts significantly inhibit pancreatic α-amylase and lipase, and intestinal α-glucosidase in vitro. The pancreatic α-amylase test exhibited an inhibition with *IC*_50_ values of 16.36 ± 1.24 µg/mL, 19.33 ± 1.40 µg/mL, 23.53 ± 1.70 µg/mL, and 17.06 ± 9.91 µg/mL for EAq, EDm, EET, and EHx, respectively. EET has the highest inhibitory capacity for intestinal α-glucosidase activity, with an *IC*_50_ of 32.23 ± 3.26 µg/mL. The extracts inhibit porcine pancreatic lipase activity, demonstrating their potential as lipase inhibitors. Specifically, at a concentration of 1 mg/mL, the highest inhibition rate (77%) was observed for EDm. To confirm these results, the inhibitory effect of these extracts on enzymes was tested in vivo. The oral intake of aqueous extract markedly reduced starch- and sucrose-induced hyperglycemia in healthy rats. Administration of the ethanolic extract at a specific dose of 150 mg/kg significantly reduced postprandial glycemia compared with the control group. It is, therefore, undeniable that cannabis extracts represent a promising option as a potentially effective treatment for type 2 diabetes.

## 1. Introduction

**Figure 1:**
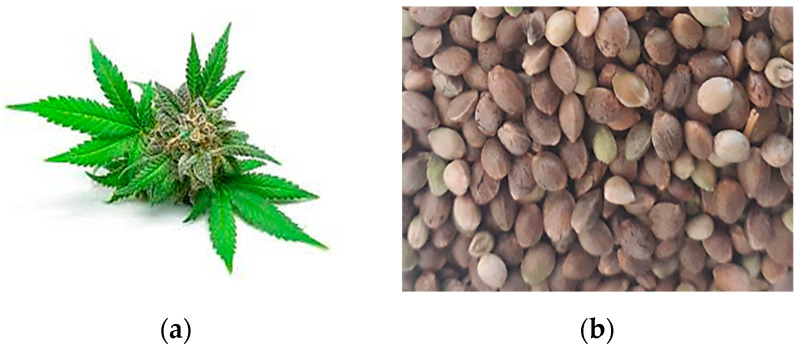
(**a**) *Cannabis sativa* L. plant; (**b**) *Cannabis sativa* L. seeds [26].

*Cannabis sativa* L. belongs to the *Cannabinaceae* family and is recognized as a significant herb with noteworthy dietary value (Figure 1). Over the past few years, numerous countries have granted permission to cultivate and process hemp varieties as an ingredient within the agri-food sector. Notably, hemp seeds remain a viable food source [1]. Cultivating cannabis is relatively low-maintenance, is renowned for its potent psychoactive properties, and is now only grown legally. This versatile plant has a rich history, having been cultivated in ancient times for various purposes, such as making clothing, ropes, and sails [2]. Arab traders introduced hemp from India to Africa, where it found valuable applications for addressing health issues, including dysentery, asthma, malaria, and fever [3]. Understanding the biochemistry of hemp holds significance, as research indicates its growing importance as a sustainable raw material. It provides a strong foundation for future industrial and agricultural advancements, with the potential for further utilization and modifications. This plant and its derivatives have long been used for their calming, analgesic, narcotic, antispasmodic, and other therapeutic properties [4]. It can be used to treat conditions such as migraines, photophobia, hemorrhoids, and asthma. In addition, cannabis can lower blood pressure through vasodilation, increase heart rate, and stimulate appetite; however, it also produces certain side effects, such as dizziness and dry mouth [5]. This plant contains analgesic, anti-inflammatory [6], and antimicrobial activity [7]. Many phytocannabinoids have an anticancer effect in vitro and in vivo, demonstrating their potential efficacy against different forms of cancer, including breast, skin, prostate, lung, and glioma cells [8,9,10]. *Cannabis* synthesizes a diverse range of compounds, encompassing over 150 phytocannabinoids, a wide variety of terpenes, as well as cannflavins characterized by geranylated (C10) [11,12], and prenylated (C5) flavones [13]. Hemp seeds contain a variety of biologically active secondary metabolites. Among these, polyphenols (mainly flavonoids, stilbenes, and lignanamides), alkaloids, cannabinoids, and terpenoids are the most characteristic of cannabis seeds [14,15]. These compounds have preventive effects on various diseases, including oxidative stress, cancer and hypertension [14,16]. Hemp seeds are rich in highly digestible protein, accounting for 31% of their content [17]. Other products, including extracts, have produced syrups and flavors from *Cannabis* teas [18]. Thus, as a food supplement with potential therapeutic effects, *Cannabis sativa* L. seeds can be used in particular for the treatment of hypertension due to their polyphenol content. *Cannabis* has a long history in Africa, where it has been used recreationally and in traditional medicine for centuries; having been introduced by Arab traders from India, in traditional African medicine, cannabis has been employed to address a broad spectrum of illnesses, covering over 20 different conditions, including diabetes [19]. This plant has antioxidant, anti-cancer, neuroprotective [20], anti-diabetic, and anti-inflammatory properties [21]. It possesses significant promise for use in addressing infectious diseases.

Diabetes mellitus, among the most prevalent conditions globally, is a chronic metabolic disease characterized by hyperglycemia and carbohydrate, protein, and lipid metabolism disorders. The occurrence of diabetes is increasing, with the number of cases anticipated to reach 552 million by 2030 [22]. Diabetes mellitus is a persistent metabolic condition characterized by elevated blood glucose fluctuations and disruptions in the metabolism of lipids, carbohydrates, and proteins [23]. Among those identified with type 2 diabetes mellitus, attaining typical blood glucose levels necessitates the use of oral hypoglycemic agents or insulin. Nevertheless, these medications have demonstrated restricted effectiveness and are associated with unwanted side effects. This has prompted growing attention to be directed toward herbal remedies as a means to mitigate the adverse effects [24,25]. Undesirable side effects have led to the use of plants as alternatives [24,25]. Building upon these investigations, we expanded our research by employing alternative extraction methodologies, diverse solvents, and innovative techniques to further explore this plant’s potential anti-diabetic properties.

Previous studies have described these compounds’ chemical composition and certain biological activities. Nonetheless, the potential in vivo and in vitro antidiabetic properties of *Cannabis sativa* L. seeds remain unexplored. In that study, we identified the phenolic compounds of the extract by HPLC-DAD. In addition, we studied the inhibitory activity of *C. sativa* L. seed extracts against pancreatic α-amylase and intestinal α-glucosidase. Various methods have been used in vivo and in vitro, including assessing its antihyperglycemic effect.

## 2. Results

### 2.1. High-Performance Liquid Chromatography HPLC

**Figure 2:**
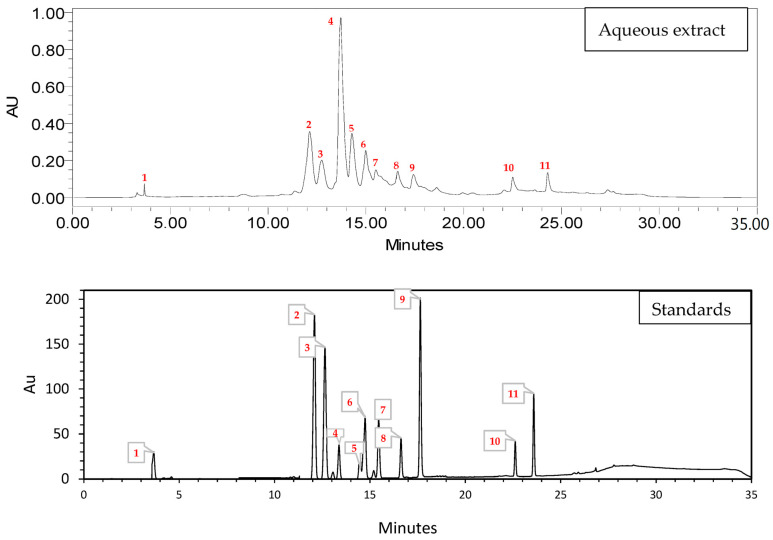
HPLC chromatogram of the *Cannabis sativa* L. seed aqueous extract (catechin 1; 4-hydroxybenzoic acid 2; vanillin 3; 3-hydroxycinnamic acid 4; rutin 5; p-coumaric acid 6; quercetin 7; luteolin 8; quercetin 3-*O*-β-d-glucoside 9; kaempferol 10; flavone 11).

The HPLC analysis results for phenolic compounds in *Cannabis* seed extract (Table 1 and Figure 2) showed that 3-hydroxycinnamic acid had the highest % area at 38.08%, indicating that it is the most abundant phenolic compound in the extract. 4-Hydroxybenzoic acid and rutin were also present in significant amounts, with % area values of 17.89% and 11.51%, respectively. P-coumaric acid, vanillin, and quercetin 3-*O*-β-d-glucoside are present at moderate levels, ranging from 5.21% to 8.49%. Quercetin and flavone are in relatively lower amounts, with % area values of 5.21% and 2.24%, respectively. Catechin, luteolin, and kaempferol were found in trace amounts, with % area values < 2%. The HPLC analysis indicated that the most abundant phenolic compound in the *cannabis* seed extract was 3-hydroxycinnamic acid, followed by 4-hydroxybenzoic acid and rutin. These results reveal important information about the composition of phenolic compounds in the extract, which can have implications for potential health benefits and applications.

Furthermore, analysis of the phenolic profile of *Cannabis* seeds using HPLC-DAD revealed a preponderance of hydroxycinnamate [27]. According to Chang et al., p-coumaric acid is a primary phenolic component in the EA fraction [28]. Similar work by Haddou et al. showed that the main components of the aqueous extract are 4-hydroxybenzoic acid, vanillic acid, and vanillin [21]. Incorporating polyphenol-rich foods into a balanced diet, including fruits, vegetables, and certain teas, can help with overall health and blood sugar control. Many of these substances contribute to their potential for preventing chronic ailments, such as cardiovascular diseases, inflammation, cancer, and diabetes [20]. The various phenolic compounds in these extracts can significantly impact their biological functionality, including the quantity and positioning of hydroxyl groups within aromatic rings, affecting their toxicity to microorganisms. It is important to note that antimicrobial effectiveness does not solely hinge on the presence of phenolic compounds but rather encompasses various other secondary metabolites. A potential association has been suggested between the amount of phenolic components and their capacity to hinder the functions of α-glucosidase and α-amylase enzymes [29,30,31,32].

### 2.2. Oral Glucose Tolerance Test (OGTT)

#### 2.2.1. Effect of EAq

**Figure 3:**
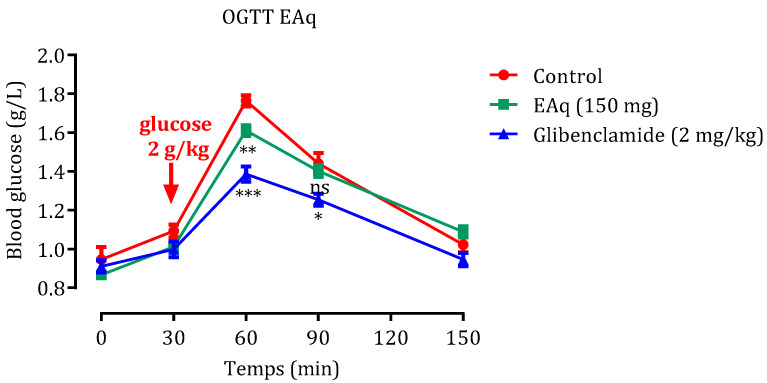
Impact of EAq and glibenclamide on post-meal blood sugar levels in healthy rats following glucose overload (2 g/kg). The data are expressed as means ± SEM (*n* = 6). Significance levels are represented as *** *p* < 0.001, ** *p* < 0.01, and * *p* < 0.05 in comparison to the group under standard conditions. ns: no significance.

OGTT examination was performed to assess the body’s glucose tolerance; the findings are depicted in Figure 3. In normal rats subjected to an OGTT, introducing 2 g/kg of glucose led to an initial glycemia level of 0.92 g/L in the group treated with distilled water alone (control group). Postprandial hyperglycemia after 60 min peaked at 1.76 ± 0.03 g/L and then gradually decreased after 150 min to an average of 1.02 ± 0.08 g/L. However, after 60 min, administering 150 mg/Kg EAq markedly inhibited the post-meal increase in blood sugar compared with the control group (*p* < 0.01). In addition, compared to the control group, glibenclamide significantly decreased blood glucose levels after 60 and 90 min (*p* < 0.001, *p* < 0.05, respectively).

#### 2.2.2. Effect of EET

**Figure 4:**
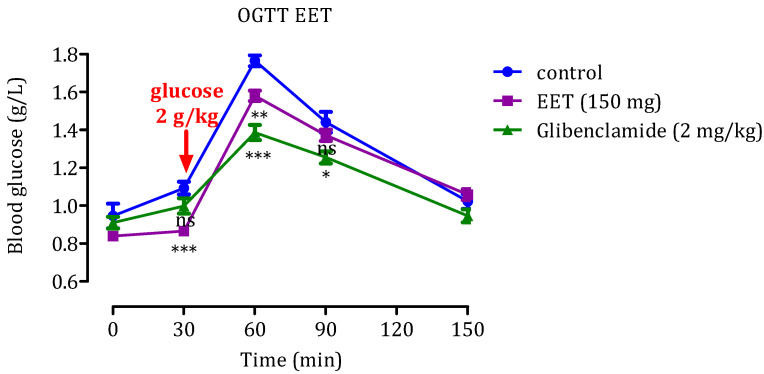
Impact of EET and glibenclamide on postprandial blood glucose level in healthy rats following glucose overload (2 g/kg). The values denote the means ± SEM (*n* = 6). Statistical significance is denoted as *** *p* < 0.001, ** *p* < 0.01, and * *p* < 0.05 when compared to the group under standard conditions. ns: no significance.

Findings from the glucose tolerance examination on healthy rats using a 2 g/kg glucose concentration are depicted in Figure 4. Within the group under standard conditions, exclusively administered distilled water, the initial blood sugar level was recorded at 0.94 g/L. Following the administration of glucose, the rats encountered elevated post-meal blood sugar levels, peaking at 1.76 ± 0.03 g/L after 60 min. Afterward, the levels gradually decreased, averaging 1.02 ± 0.08 g/L after 150 min. Notably, upon treatment with EET administered at a rate of 150 mg/kg, postprandial hyperglycemia concentrations were significantly reduced. Statistically significant suppression was observed after 30 and 60 min (*p* < 0.001, *p* < 0.01, respectively).

Additionally, the administration of glibenclamide led to a significant reduction in blood glucose levels after 60 min (*p* < 0.001) and 90 min (*p* < 0.01) when compared to the control group. Integrating the results obtained from in vitro and in vivo investigations provides a more robust and reliable basis for drawing conclusions and making informed decisions about using these substances in diabetes management and potential drug development. Based on these conclusive and compelling results, it is undeniable that cannabis extracts represent a promising, potentially effective treatment for type 2 diabetes. These significant findings pave the way for new opportunities in medical research, highlighting the prominent role that cannabis compounds can play in managing and improving medical conditions related to this disease.

### 2.3. In Vitro Inhibition of Pancreatic α-Amylase and Lipase and Intestinal α-Glucosidase Enzymatic Activities

#### 2.3.1. In Vitro, α-Amylase Inhibitory Activity

**Figure 5:**
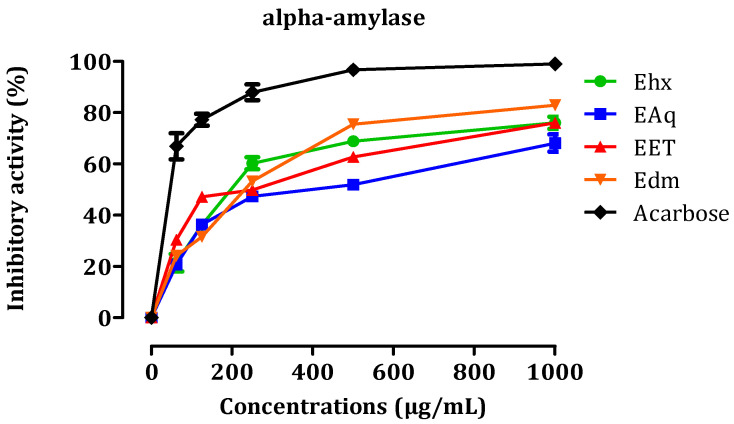
In vitro inhibitory effects of pancreatic α-amylase by EHx, EAq, EET, EDm, and acarbose. The presented values represent the means ± SEM.

Diabetes is a long-term medical condition that arises when the pancreas fails to produce adequate insulin or when the body cannot utilize the insulin it produces effectively. Antidiabetic activity is commonly evaluated by measuring the inhibition of α-amylase. Figure 4 presents the results of the inhibitory effects of EHx, EAq, EET, EDm, and acarbose on this enzyme in vitro. We assessed the inhibitory effects of various concentrations (0.062, 1, 1.5, and 1 mg/mL) of four distinct *C. sativa* L. extracts, namely EAq, EHx, EDm, and EET, on pancreatic α-amylase. The results revealed that these extracts exhibited significant inhibitory activity against α-amylase. Comparing these findings to the chemical composition of each extract provides insights into the potential compounds responsible for their inhibitory effects. First, EAq exhibited the most potent inhibitory activity with the lowest *IC*_50_ value of 16.36 ± 1.24 µg/mL. It may owe its effectiveness to specific compounds in its composition (Figure 5). The chemical composition of EAq includes catechin di-hydrate, cinnamic acid, p-coumaric acid, caffeic acid, benzoic acid, and sinapinic acid, which could contribute to its α-amylase inhibitory activity [26].

EHx, with an *IC*_50_ value of 17.06 ± 9.91 µg/mL, follows closely regarding inhibitory potency. The composition of EHx includes palmitic acid methyl ester, linoleic acid methyl ester, 7-octadecenoic acid methyl ester, linolenic acid methyl ester, stearic acid methyl ester, and heptacosanoic acid methyl ester [26], which likely contribute to its inhibition of α-amylase activity. EDm, with an *IC*_50_ value of 19.33 ± 1.40 µg/mL, and EET, with an *IC*_50_ value of 23.53 ± 1.70 µg/mL, exhibited relatively lower inhibitory potency compared to EAq and EHx. The chemical composition of EDm, which is obtained through dichloromethane extraction, includes catechin acid di-hydrate, 6 hydroxy flavone, ferulic acid, 8 methoxy flavone, hesperidin acid, and rutin. Meanwhile, EET, extracted with ethanol, contains naringin acid as the major component, along with rutin, hesperidin acid, o-dianisidine, ferulic acid, chlorogenic acid, coumarin, and cinnamic acid. These findings suggest that among the tested *C. sativa* L. extracts, EAq exhibited the most potent inhibitory activity against α-amylase, followed closely by EHx, with EDm and EET showing relatively lower inhibitory potency in decreasing order of effectiveness. 

The primary synthesis of α-amylase occurs in the pancreas, where it breaks down complex carbohydrates, such as starch, into simpler sugars (monosaccharides) during digestion. These monosaccharides, particularly glucose, are further broken down by another enzyme called α-glucosidase into glucose molecules. After absorption in the digestive system, glucose enters the bloodstream, increasing blood sugar levels. The enzyme under investigation, pancreatic α-amylase, breaks down polysaccharides into disaccharides and oligosaccharides. When our extracts inhibit the activity of this enzyme, a reduction occurs in the formation of oligosaccharides and disaccharides. As a result, glucose absorption into the bloodstream is decreased. *C. sativa* L. has demonstrated potential as an agent with antidiabetic activity [33]. The essential oil of cannabis has also been evaluated against α-amylase, however, it showed no significant effects [34].

#### 2.3.2. In Vitro Pancreatic Lipase Inhibitory Effect

Obesity denotes a state where the body accumulates surplus fat, which can elevate the risk of several ailments, including diabetes [35]. Obesity is characterized by the accumulation of surplus fat within the body. Reducing body weight can be achieved by impeding fat absorption, which involves suppressing pancreatic lipase activity. This enzymatic factor is pivotal in the fat metabolism process [36]. Lipase is a crucial enzyme contributing to the breakdown of triglycerides into monoacylglycerols and fatty acids within the gastrointestinal tract [37]. Numerous variations of lipases exist, with one category being pancreatic lipases. These specific enzymes are responsible for approximately 50–60% of the fat absorption process [38]. Currently, the sole pancreatic lipase inhibitor accessible for clinical use is orlistat, derived from Lipstatin, which originates as an irreversible pancreatic lipase inhibitor in *Streptomyces* bacteria [39].

**Figure 6:**
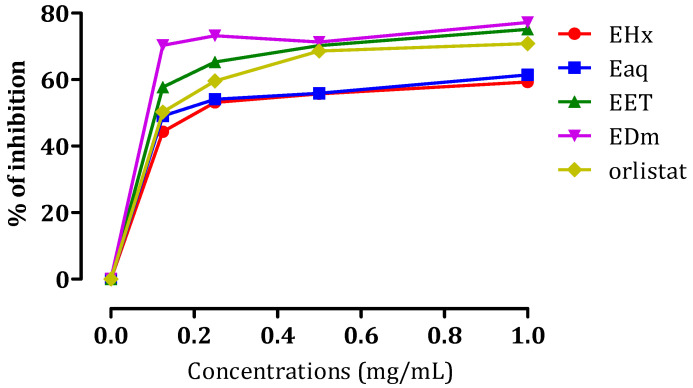
Porcine pancreatic lipase inhibitory activity of EHx, EAq, EET, EDm, and orlistat. Data are presented as mean ± SD (*n* = 3).

However, alternative approaches involving active compounds derived from natural sources have undergone extensive investigation as potential remedies for obesity. The aim is to develop more efficacious treatments with fewer associated side effects [40]. As a segment of this research, extracts obtained from *C. sativa* L. were analyzed to determine their potential to act as inhibitors of porcine pancreatic lipase. The results presented in Figure 6 demonstrate the inhibitory effects of EHx, EAq, EET, and EDm on pancreatic lipase activity through the p-nitrophenyl palmitate-based assay. These extracts exhibited inhibitory activity against porcine pancreatic lipase activity, demonstrating their potential as lipase inhibitors. Specifically, at a 1 mg/mL concentration, EHx displayed a 59% inhibition rate, EAq exhibited a 61% inhibition rate, EEt demonstrated a more substantial 75% inhibition rate, and EDm presented the highest inhibition rate at 77%. To quantitatively assess the potency of these inhibitory effects, *IC*_50_ values were calculated, representing the concentration of each extract needed to achieve a 50% inhibition of porcine pancreatic lipase activity. The determined *IC*_50_ values for EHx, EAq, EET, and EDm were 0.202 ± 0.0505, 0.170 ± 0.005, 0.109 ± 0.0006, and 0.089 ± 0.001 mg/mL, respectively. These values offer valuable insights into the relative strengths of the inhibitory properties of the extracts. For comparative purposes, the *IC*_50_ value of the reference compound orlistat, a known lipase inhibitor, was measured at 0.128 ± 0.003 mg/mL. This reference value served as a benchmark to evaluate the effectiveness of the extracts’ inhibitory activities against pancreatic lipase.

In terms of relating these findings to the chemical composition of the extracts, the EHx contains major components, including naringin acid, rutin, and hesperidin acid. EDm is primarily rich in catechin acid di-hydrate, which may account for its high inhibitory rate. EAq contains various bioactive compounds, such as catechin di-hydrate, O. dianisidine, cinnamic acid, p-coumaric acid, caffeic acid, benzoic acid, and sinapinic acid. In addition, the major component in EET is naringin acid, constituting approximately 41.92% of the extract. The combined presence of these compounds could collectively contribute to its lipase inhibitory potential. In another study, the essential oil derived from *C. sativa* demonstrated the ability to inhibit pancreatic lipase activity, achieving a 70.14 ± 2.40 mg orlistat equivalent per gram of oil [34]. In a separate study, the aqueous extract was ineffective as a lipase inhibitor [41].

#### 2.3.3. In Vitro α-Glucosidase Inhibitory Effect

**Figure 7:**
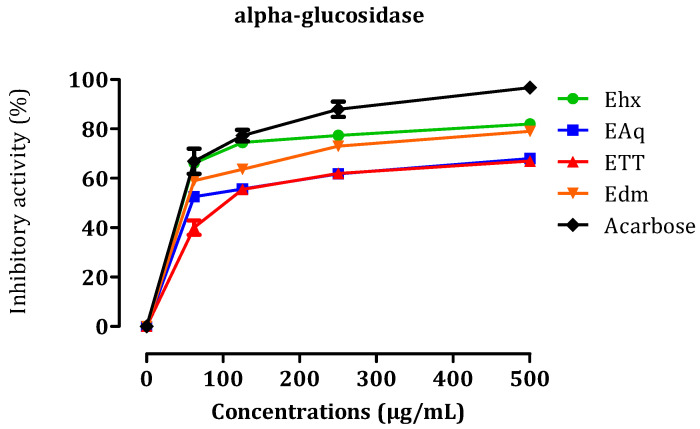
Inhibition of α-glucosidase by EHx, EAq, EET, EDm, and acarbose in vivo. The results are presented as the means ± SEM.

In this in vitro study, the inhibitory effects of different doses (0.062, 0.25, 0.5, 1 mg/mL) of *C. sativa* L. extracts, including EET, EAq, EDm, and EHx, on intestinal α-glucosidase activity were investigated (Figure 7). The calculated *IC*_50_ values for these inhibitory activities were as follows: EET, 32.23 ± 3.26 µg/mL; EAq, 41.93 ± 0.33 µg/mL; EDm, 47.6 ± 0.69 µg/mL; EHx, 52.16 ± 0.55 µg/mL, with acarbose, a known α-glucosidase inhibitor, exhibiting an *IC*_50_ of 52.5 ± 2.67 µg/mL. These results suggest that *C. sativa* L. extracts, notably EET, display significant inhibitory efficacy on α-glucosidase compared to acarbose, highlighting their potential as alternatives for diabetes treatment with potentially fewer side effects. The chemical compositions of these extracts, including naringin acid, catechin acid di-hydrate, and other bioactive compounds, may contribute to their observed inhibitory effects. However, further research is required to elucidate the precise mechanisms responsible for the associated therapeutic effects. The in vitro study conducted by Suttithumsatid et al. [42] on α-glucosidase demonstrated that cannabidiol and tetrahydrocannabinol exhibit promising inhibitory activity, surpassing that of acarbose. These two molecules could be responsible for the anti-diabetic effect of cannabis extracts. An in vivo study is necessary to confirm or invalidate the in vitro results.

### 2.4. Inhibition of Pancreatic α-Amylase and Intestinal α-Glucosidase In Vivo

#### 2.4.1. Effect of the EAq

**Figure 8:**
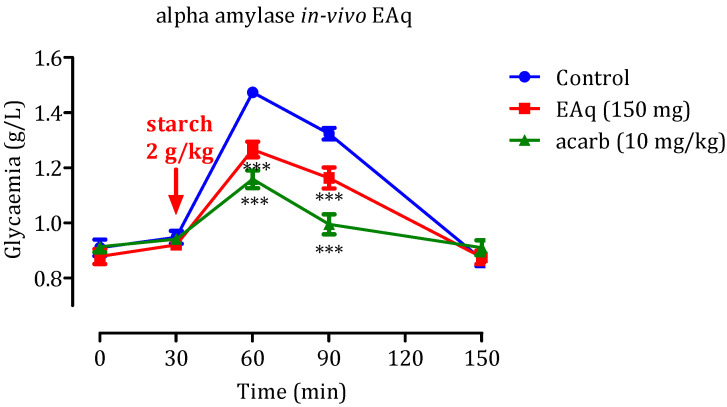
Impact of EAq and acarbose on blood glucose levels following starch overload (2 g/kg) in normal rats (*n* = 6), *** *p* < 0.001 statistical significance was observed compared to the control group.

Herbal medicines have become indispensable due to the intensification of research in traditional medicine. Approximately 800 plants are estimated to possess anti-diabetic properties [43], the activity of which can be assessed by in vivo analyses. The results of our in vivo assessment are provided in Figure 8. Following starch administration at 2 g/kg, a significant increase in glycemia rates was observed, reaching a zenith of 1.49 ± 0.02 g/L. Over the next 60 min, the glycemia gradually decreased to 1.32 ± 0.07 g/L. However, when EAq (150 mg/kg) was administered, a significant reduction in postprandial glycemia was observed (*p* < 0.001 at 30 min and *p* < 0.001 at 60 min). The blood glucose values at these time points were 1.25 ± 0.06 g/L and 0.13 ± 0.004 g/L, respectively, relative to the control group. Notably, no statistically significant distinction was observed in glycemia at 150 min after EAq administration. Furthermore, administering acarbose at 10 mg/kg suppressed the hyperglycemia provoked by starch (*p* < 0.001 at 30 and 60 min). The blood glucose values at these time points were 1.11 ± 0.06 g/L and 0.95 ± 0.004 g/L, respectively. These findings suggest that EAq and acarbose have potent dose-dependent inhibitory effects on postprandial glycemia. Acarbose, in particular, demonstrated stronger inhibitory activity than EAq. Overall, the results indicate that EAq and acarbose have potential as antidiabetic agents, with EAq significantly reducing postprandial glycemia. However, acarbose exhibited even more robust inhibitory effects against starch-induced hyperglycemia.

The significant variation in results can be attributed to the use of selected solvents. Indeed, the multiple extracts present different phytochemical compositions and bioactive compounds. These variations may influence enzyme inhibitory activity and explain why some extracts showed greater efficacy than others.

#### 2.4.2. Effect of the EET

**Figure 9:**
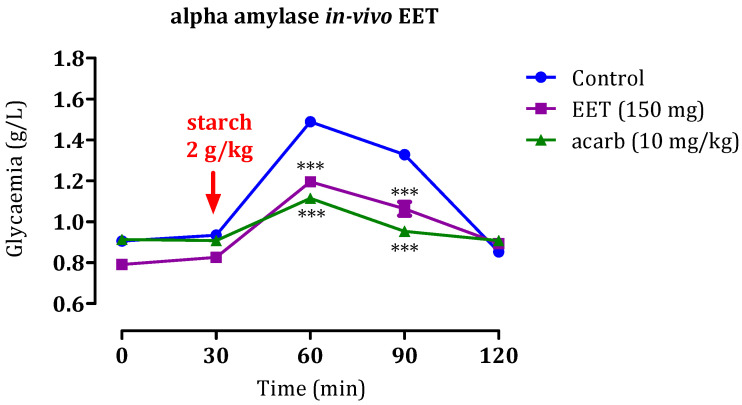
Impact of EET and acarbose on blood glucose levels following an excess of starch (2 g/kg) in normal rats (*n* = 6). *** *p* < 0.001 compared to the control group.

Administration of EET (150 mg/kg) significantly reduced postprandial glycemia at 60 min and 90 min (Figure 8; *p*-value < 0.001) with blood glucose values of 1.19 ± 0.02 and 1.06 ± 0.003 g/L, respectively, in contrast with the control group. Nevertheless, at 120 min, no notable distinction was observed in glycemia between the EEt-administered and control groups. These findings indicate that, like acarbose, EEt possesses strong dose-dependent inhibitory effects on postprandial glycemia. The results show that EET administration at 150 mg/kg substantially reduces blood glucose levels at 60 and 90 min after starch intake, indicating its potential as an effective antidiabetic agent during the early postprandial period (Figure 9). However, the effect seems to diminish by 120 min, suggesting that further investigation may be needed to explore the duration and optimal dosing for sustained antidiabetic effects.

### 2.5. In Vivo α-Glucosidase Inhibitory Effect

#### 2.5.1. Effect of the EAq

**Figure 10:**
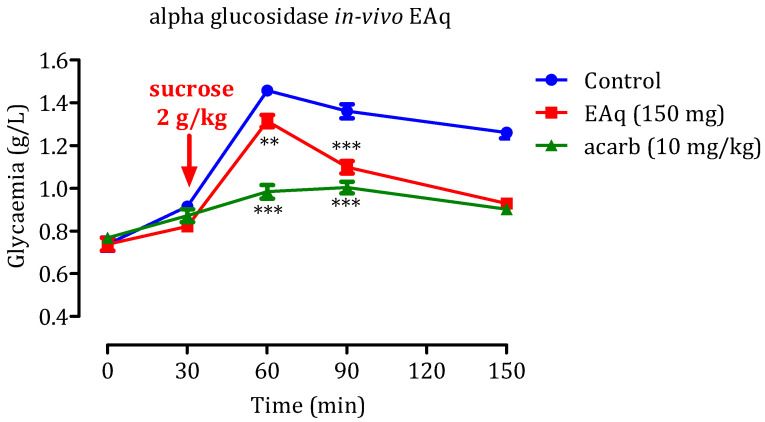
Impact of EAq and acarbose on blood glucose levels following an excess of sucrose (2 g/kg) in healthy rats (*n* = 6): Statistical significance (** *p* < 0.01 and *** *p* < 0.001) was noted in comparison to the control group.

Administering sucrose (2 g/kg) led to a significant increase in postprandial glycemia (Figure 10), resulted in blood glucose levels reaching 1.45 ± 0.03 g/L at 60 min and 1.36 ± 0.01 g/L at 90 min. However, when EAq (150 mg/kg) was administered, postprandial glycemia was remarkably reduced. The decrease in blood glucose values was statistically significant at 60 min (1.31 ± 0.02 g/L; *p* < 0.01) and 90 min (*p* < 0.001; 1.01 ± 0.04 g/L), relative to the control group. Similarly, 10 mg/kg acarbose effectively reduced hyperglycemia induced by sucrose (*p* 0.001) at 60 and 90 min, with blood glucose levels of 0.98 g/L and 1 g/L, respectively. That suggests that EAq and acarbose can potentially reduce postprandial glycemia dose-dependently. EAq, at a dose of 150 mg/kg, demonstrated a considerable decrease in glycaemia rates at 60 and 90 min after sucrose intake. Similarly, at a dosage of 10 mg/kg, acarbose showed robust inhibitory effects against sucrose-induced hyperglycemia simultaneously. These results indicate that EAq and acarbose may be beneficial in managing postprandial hyperglycemia, making them potential candidates as antidiabetic agents for controlling blood glucose levels after meals. Further research and clinical studies are necessary to fully understand their mechanisms of action and evaluate their long-term efficacy and safety in diabetes management.

#### 2.5.2. Impact of EET

**Figure 11:**
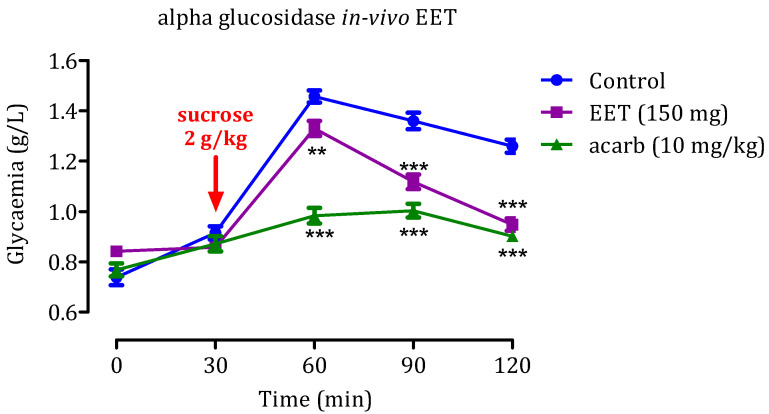
Impact of EET and acarbose on blood glucose levels following an excess of sucrose (2 g/kg) in healthy rats (*n* = 6); Significance levels (** *p* < 0.01 and *** *p* < 0.001) were noted compared to the control group.

The results of administering EET at 150 mg/Kg demonstrate a significant reduction in postprandial glycemia compared with the control group (*p* < 0.01 at 60 min, *p* < 0.001 at 90 min, and *p* < 0.001 at 150 min; Figure 11). The observed glycaemia levels were 1.33 ± 0.01 g/L, 1.11 ± 0.05 g/L, and 0.94 g/L at 60, 90, and 150 min, respectively. This suggests that EET may effectively manage postprandial hyperglycemia during the early and late post-meal periods. Hence, EET and acarbose hold promise as substances for decreasing postprandial blood glucose levels in a dose-dependent manner.

In various scientific studies, observations have revealed variations in α-glucosidase and α-amylase inhibition depending on the specific nature of the enzymatic inhibitors in plant extracts. Previous research suggests the existence of a potential correlation between the properties of phenolic compounds and their ability to modulate the activity of α-glucosidases and α-amylases [30]. P-coumaric acid (p-CA) from *Cannabis sativa* demonstrated the potential to protect against diabetes in rats. It effectively lowered blood glucose levels, reduced gluconeogenic enzyme activities, increased insulin levels, and improved glucose and lipid metabolism. P-CA also showed beneficial effects on cholesterol levels and significantly decreased expression of GLUT2 mRNA in the pancreas. These findings highlight p-CA as a promising candidate for addressing metabolic disorders [44]. In diabetic rats, oral administration of 4-hydroxybenzoic acid led to a dose-dependent reduction in plasma glucose levels, complementing the positive effects of p-coumaric acid from *Cannabis sativa*. These findings highlight the potential of diverse compounds in managing glucose levels and addressing metabolic disorders [45]. Rutin also demonstrates antihyperglycemic properties and protective effects against diabetic complications through various mechanisms. These include diminishing carbohydrate absorption, hindering tissue gluconeogenesis, augmenting glucose uptake, promoting insulin secretion, and preserving Langerhans islets. Furthermore, rutin reduces the generation of precursors to advanced glycation end-products. These multifaceted effects collectively contribute to rutin’s protective role against hyperglycemia and the development of diabetic complications [46].

## 3. Materials and Methods

### 3.1. Plant Materials and Extraction

**Figure 12:**
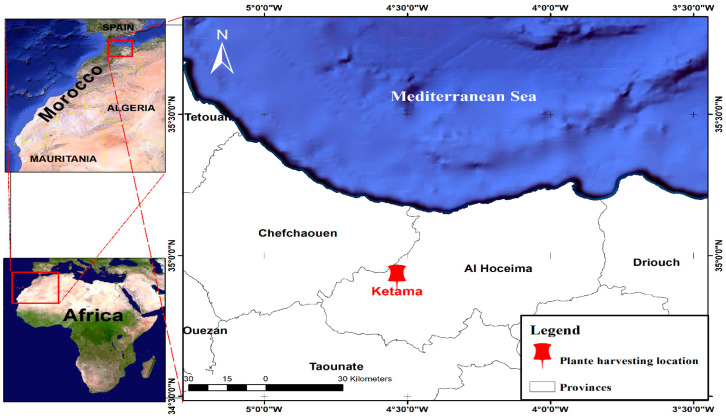
Plant harvesting location map of *C. sativa* L.

On December 2021, cannabis seeds (Figure 1) were harvested in Ketama, a town in northern Morocco that is one of the most renowned worldwide for the quality of its hemp (Figure 12). The seeds were dried for one week at room temperature (25 °C).

To prepare the *C. sativa* L. seed extract, the seeds were separated, sieved to remove suspended matter and dust and ground into a very fine powder using a grinder. Various extracts were extracted by maceration from the seed powder using the following extraction protocol: solid–liquid extraction of 65 g of crushed seeds at room temperature under magnetic stirring in the presence of 300 mL of hexane in accordance with the procedure developed by Haddou et al. [26]. The solution was vacuum-filtered through a Buchner funnel, and the filtrate was recovered and filtered through a filter crucible to obtain an acceptable filtrate. The filtrate was passed through a steam rotor to evaporate the solvent completely and collect the oil separately. The filtrate was dried and evaporated in an oven at 35 °C. Dichloromethane extract, ethanol, and aqueous extract were treated according to the same method, with a modification in the maceration time. The extracts were then stored in dark bottles at −4 °C until use. Hexane was used to remove fatty acids and nonpolar molecules; dichloromethane was used to extract moderately polar molecules, and ethanol and water were employed to extract polar compounds, such as polyphenols.

### 3.2. Experimental Animals

Wistar rats of both sexes, aged 3 months, and Swiss albino mice, aged 2 months, were utilized as the animal subjects. The rats were housed under standard laboratory conditions and provided a regular diet with unlimited access to water. These conditions included a 12-h light and 12-h dark cycle and a controlled temperature of 24 ± 2 °C. The experiment was performed at Mohammed First University, Faculty of Sciences, Oujda, Morocco.

All procedures involving the animals strictly adhered to the guidelines outlined in the National Institutes of Health’s Guide for the Treatment and Use of Laboratory Animals [47]. The study was conducted in full compliance with an approved protocol, overseen by the Institutional Care and Use Committee at the Faculty of Sciences in Oujda (certification number: 15/19-LBBEH-06).

### 3.3. High-Performance Liquid Chromatography Analysis

The phenolic compounds contained in the ethanolic extract were analyzed by high-performance liquid chromatography (HPLC) with an Agilent 1200 system (Agilent Technologies, Palo Alto, CA, USA) connected to a diode array UV detector from Bruker (Germany). In this analytical process, a 20 μL volume of each extract was injected into a Zorbax XDB-C18 column (5 µm porosity, 250 × 4.6 mm), part of the Agilent Technologies 1100 series system (Palo Alto, CA, USA). The following gradient was applied to elute the samples: 0–15 min with 20% mobile phase B (methanol) followed by 15–25 min with 100% B, and 25–35 min with 20% B. The mobile phases used for elution comprised A (water/0.5% acetic acid) and B (methanol), and the elution process occurred at a consistent flow rate of 1.69 mL/min, with a constant temperature of 25 °C. Spectrophotometric detection was conducted at 280 nm. The compounds were identified by comparing their retention times and UV spectra with those of established standards. The chromatographic data were analyzed according to the strategy provided by Loukili et al. [48,49].

### 3.4. Antihyperglycemic Effect of C. sativa L.

#### Oral Glucose Tolerance Test

The study evaluated the antihyperglycemic effects of *C. sativa* L. in normal rats by administering aqueous and ethanolic extracts. The experimental protocol was conducted as defined by hbika et al. [50] with slight adjustments. Before the experiment, the rats were subjected to a fasting period of 16 h with only water available and were arbitrarily assigned to six groups: the control group received distilled water through intragastric gavage (i.g.); the second group received EAq or EET extract (150 mg/kg), while the last group received glibenclamide (2 mg/kg) diluted with distilled water. After 30 min, rats received an oral administration of glucose (2 g/kg), and blood samples were obtained from the end of the rat’s tail after 30, 60, 90, and 120 min. The rats were sedated with a mild ethanol solution and positioned in a cage alongside anesthesia-soaked cotton for 2–3 min to minimize pain and discomfort during the procedures. The collected blood underwent centrifugation, and glucose levels were measured utilizing the glucose oxidase–peroxidase method (GOP–POD) with a Hermle Z230H centrifuge (Gosheim, Germany) and glucose, SGM Italia reagents.

### 3.5. In Vitro Inhibition of Carbohydrate Hydrolase Enzymes

#### Inhibition of Pancreatic α-Amylase Assessed In Vitro

Ouahabi et al. established a strategy for investigating the in vitro inhibition on pancreatic α-amylase [51] with certain adjustments. The experimental mixtures consisted of 200 µL of a phosphate buffer solution (0.02 M; pH = 6.9) and 200 µL of an α-amylase enzyme solution. Before 10 min incubation at 37 °C, 200 µL of each EAq, EDm, EET, and EHx solutions (0.062, 0.125, 0.25, 0.5, 1 mg/mL) were added prior to the addition of 200 µL of a 1% starch solution to each tube, followed by incubation at 37 °C for 15 min. To halt the enzymatic process, 600 µL of 3,5-dinitrosalicylic acid (DNSA) reagent was included, and the tubes were subsequently placed in an incubation chamber at 100 °C for 8 min. Following the thermal shock, the tubes were placed in an ice-water bath. Subsequently, 1 mL of distilled water was introduced to each tube to dilute the mixtures. The spectrophotometer was employed to measure the absorbance at a wavelength of 540 nm. A blank reaction was generated by replacing the extract with 200 μL of phosphate buffer to establish 100% catalytic function. Additionally, a blank reaction without the enzyme solution was created using the plant extract at each dose. Acarbose, employed as a positive control at equivalent concentrations, underwent a reaction similar to that of the plant extract. The inhibition ratio was calculated using the following equation:

$$Inhibition\left(\%\right)=\frac{A_{control}-A_{sample}}{A_{control}}\times 100$$

A_control_: Absorption in relation to enzymatic activity in the absence of an inhibitor;

A_sample_: Absorbance in relation to enzymatic activity in the presence of extracts or acarbose.

The *IC*_50_ (Concentration of sample causing a 50% inhibition of α-amylase enzyme activity) was graphically established by employing the function: percentage of inhibition = f(log(sample concentration)).

### 3.6. Inhibition of Intestinal α-Glucosidase Assessed In Vitro 

The inhibitory activity for α-glucosidase was determined using the protocol described by Elrherabi et al. [52], with slight modifications. The test mixtures comprised 1 mL of phosphate buffer solution (pH = 7.5), 0.1 mL of a α-glucosidase enzyme (10 IU) solution, and 200 µL of EAq, EET, EDm, or EHx (0.062, 0.125, 0.25, 0.5, 1 mg/mL). As control samples, an equal volume of distilled water and acarbose (0.062, 0.125, 0.25, 0.5, 1 mg/mL) were utilized as negative and positive controls, respectively. After pre-incubation at 37 °C for 20 min, 0.1 mL of sucrose was introduced to each tube, followed by a 5-min incubation at 100 °C to stop the reaction. Subsequently, following the addition of 1 mL of GOD–POD (glucose oxidase–peroxidase), the mixture was further incubated at 37 °C for 10 min. The absorbance of the mixture was subsequently measured at a wavelength of 500 nm with a spectrophotometer. Subsequently, the inhibition ratio was calculated using the following equation:

$$Inhibition\left(\%\right)=\frac{A_{control}-A_{sample}}{A_{control}}\times 100$$

A_control_: Absorption related to enzymatic activity effect in the absence of an inhibitor;

A_sample_: Absorption associated with enzymatic activity in the presence of extract or acarbose.

The *IC*_50_ (concentration of the sample causing a 50% inhibition of α-amylase activity) was graphically established by employing the function: percentage of inhibition = f(log(sample concentration)).

#### Pancreatic Lipase Inhibitory Test

The pancreatic lipase inhibition assay was adapted from the methodology outlined by Jaradat et al. [53]. For this particular investigation, the substrate of choice was p-nitrophenyl palmitate (pNPP). To prepare the stock solution, the plant extract was dissolved in Tris HCl buffer (pH 8) until it reached a concentration of 1000 µg/mL. Subsequently, different concentrations were created via serial dilution (0.125, 0.25, 0.5, and 1 mg/mL). The porcine pancreatic lipase solution was created by dissolving 10 mg of the enzyme in 10 mL of Tris HCl buffer at pH 8.

Additionally, a stock solution of the substrate p-nitrophenyl palmitate (pNPP) was made by dissolving 0.19 g of pNPP in 10 mL of acetonitrile. A mixture was formed to conduct the inhibition activity test by combining 0.1 mL of lipase at 1 mg/mL, 0.2 mL of the plant extract solution at various concentrations, and 0.7 mL of Tris HCl buffer at pH 8. The resulting mixture was thoroughly mixed and incubated at 37 °C for 15 min. After incubation, 0.1 mL of the substrate solution (pNPP) was added to the mixture, followed by an additional 30-min incubation at 37 °C. The absorbance of the solution was measured using a UV-visible spectrophotometer at a wavelength of 410 nm.

For comparative purposes, orlistat was employed as a positive control, and the same procedure was repeated for orlistat using the same concentrations mentioned above. To ensure precision and reliability, all experiments were conducted in triplicate. The percentage of inhibition was calculated using the subsequent equation:

$$Inhibition\left(\%\right)=\frac{\left(A0-A\right)}{A0}\times 100$$

A0: reaction mixture’s enzyme activity (absorbance) without the inhibitor (control);

A: reaction mixture’s enzyme activity (absorbance) with the inhibitor (sample).

The *IC*_50_ value represents the concentration of the plant extract (sample) needed to inhibit 50% of the enzyme activity. This value was determined through a process involving the creation of a graph that plots substrate concentration against the percentage of inhibition outcomes. Regression analysis is then employed to ascertain the concentration at which the enzyme activity is reduced by 50%, as outlined previously [36].

### 3.7. In Vivo Inhibition of Carbohydrate Hydrolase Enzymes

#### In Vivo Pancreatic α-Amylase Inhibitory Assay

The experimental protocol was conducted as defined by Bouhrim et al. [51,54]. This research utilized normal rats with a body weight between 180 and 200 g. The animals were fasted for 16 h before being split into three experimental groups; the healthy rats were randomly assigned, while the diabetic rats were categorized according to their glucose levels. The control group was exclusively administered distilled water (10 mL/kg) through intragastric gavage (i.g.), and the second group was administered a single oral dose of acarbose at 10 mg/kg. In contrast, the third group received a single oral dose of EAq or EET at 150 mg/kg. All groups were orally loaded with a starch solution (2 g/kg) administered 30 min after the initial dose. The glycemia concentration was subsequently assessed at different time intervals (0, 30, 60, 90, and 120 min) using the glucose–peroxidase assay.

### 3.8. Inhibition of Intestinal α-Glucosidase In Vivo

The intestinal α-glucosidase assay was adapted from the methodology outlined by Elrherabi et al. This study used normal rats with a 180–200 g weight range. Before the experiment, the rats were fasted for 16 h and divided into groups based on their glycemic levels. The healthy rats were arbitrarily grouped, while the diabetic rats were categorized into three experimental groups (*n* = 6/group). The control group was administered 10 mL/kg of distilled water. The second group was administered a single dose of acarbose at 10 mg/kg via gavage (i.g.). The third group was administered a single dose of EAq or EET at 150 mg/kg by intragastric gavage (i.g). Thirty minutes after administering the solutions, sucrose was administered at 2 g/kg. Glycemia concentrations were then measured at various intervals (0, 30, 60, 90, and 120 min) using the glucose–peroxidase method.

### 3.9. Statistical Analysis

The results were analyzed using GraphPad Prism 5 and expressed as the mean ± standard error of the mean (SEM). A one-way ANOVA test was conducted to analyze the results, and Turkey’s multiple comparison test was performed for multiple comparisons. Statistical significance was considered when *p* < 0.05. The sample size was *n* = 6, and statistically significant results were obtained by conducting statistical analyses to compare the means of distinct groups, employing suitable statistical tests.

## 4. Conclusions

The cultivation of cannabis seeds in Morocco has sparked interest in exploring their potential applications. Our research has revealed their ability, both in vitro and in vivo, to inhibit the activity of ⍺-amylase, pancreatic lipase, and intestinal ⍺-glucosidase. These enzymes play a crucial role in sugar digestion, and the observed hypoglycemic effects suggest the potential of our hemp seed extract in diabetes prevention. This effect can be explained by the presence of phenolic compounds as well as the notable antioxidant potency of the extracts, as substantiated by our prior investigations.

The results of this study show interesting anti-diabetic activity, suggesting its application in the medical field and food industry. However, further studies are needed to demonstrate the absence of toxicity, the method of use, and to understand the main bioactive components and their potential synergistic effects.

## Figures and Tables

**Table 1:** Qualitative phytochemical analysis of aqueous extract.

No.	Compound	Tr (min)	% Area
1	Catechin	3.674	0.39
2	4-Hydroxybenzoic acid	12.123	17.89
3	Vanillin	12.729	7.81
4	3-hydroxycinnamic acid	13.702	38.08
5	Rutin	14.276	11.51
6	p-Coumaric acid	14.983	8.49
7	Quercetin	15.508	5.21
8	Luteolin	16.616	1.53
9	Quercetin 3-*O*-β-d-glucoside	17.428	5.46
10	Kaempferol	22.504	1.40
11	Flavone	24.291	2.24

Tr (min): The retention time in minutes.

## Data Availability

Data are contained within the article.
